# Validation of a prognostic score for hidden cancer in unprovoked venous thromboembolism

**DOI:** 10.1371/journal.pone.0194673

**Published:** 2018-03-20

**Authors:** Luis Jara-Palomares, Remedios Otero, David Jimenez, Juan Manuel Praena-Fernandez, Carme Font, Conxita Falga, Silvia Soler, David Riesco, Peter Verhamme, Manuel Monreal, on behalf of RIETE Registry

**Affiliations:** 1 Department of Pneumonology, Medical Surgical Unit of Respiratory Diseases, Instituto de Biomedicina de Sevilla (IBiS), Centro de Investigación Biomédica en Red de Enfermedades Respiratorias (CIBERES), Hospital Universitario Virgen del Rocío, Seville, Spain; 2 Respiratory Department, Hospital Universitario Ramón y Cajal, IRYCIS, Madrid, Spain; 3 Statistics, Methodology and Research Evaluation Unit, Hospital Universitario Virgen del Rocío, Seville, Spain; 4 Department of Medical Oncology, Hospital Clínic, Barcelona, Spain; 5 Department of Internal Medicine, Consorci Hospitalari de Mataró, Barcelona, Spain; 6 Department of Internal Medicine, Hospital Olot i Comarcal de la Garrotxa, Gerona, Spain; 7 Department of Internal Medicine, Hospital Sant Pau i Santa Tecla, Tarragona, Spain; 8 Vascular Medicine and Haemostasis, University of Leuven, Leuven, Belgium; 9 Department of Internal Medicine, Hospital Universitario Germans Trias i Pujol de Badalona, Barcelona, Universidad Católica de Murcia, Spain; Institut d'Investigacions Biomediques de Barcelona, SPAIN

## Abstract

The usefulness of a diagnostic workup for occult cancer in patients with venous thromboembolism (VTE) is controversial. We used the RIETE (Registro Informatizado Enfermedad Trombo Embólica) database to perform a nested case-control study to validate a prognostic score that identifies patients with unprovoked VTE at increased risk for cancer. We dichotomized patients as having low- (≤2 points) or high (≥3 points) risk for cancer, and tried to validate the score at 12 and 24 months. From January 2014 to October 2016, 11,695 VTE patients were recruited. Of these, 1,360 with unprovoked VTE (11.6%) were eligible for the study. At 12 months, 52 patients (3.8%; 95%CI: 2.9–5%) were diagnosed with cancer. Among 905 patients (67%) scoring ≤2 points, 22 (2.4%) had cancer. Among 455 scoring ≥3 points, 30 (6.6%) had cancer (hazard ratio 2.8; 95%CI 1.6–5; p<0.01). C-statistic was 0.63 (95%CI 0.55–0.71). At 24 months, 58 patients (4.3%; 95%CI: 3.3–5.5%) were diagnosed with cancer. Among 905 patients scoring ≤2 points, 26 (2.9%) had cancer. Among 455 patients scoring ≥3 points, 32 (7%) had cancer (hazard ratio 2.6; 95%CI 1.5–4.3; p<0.01). C-statistic was 0.61 (95%CI, 0.54–0.69). We validated our prognostic score at 12 and 24 months, although prospective cohort validation is needed. This may help to identify patients for whom more extensive screening workup may be required.

## Introduction

Cancer patients are at an increased risk to develop venous thromboembolism (VTE), and VTE may also appear before the cancer has become symptomatic (thus leading to an early diagnosis of cancer) [1, 2]. The usefulness of a diagnostic workup for occult cancer in VTE patients is controversial, and current guidelines suggest that patients with unprovoked VTE should undergo a limited cancer screening including thorough medical history and physical examination, basic laboratory investigations and chest x-ray [3–7]. In a recent study using the RIETE registry database, we built a prognostic model to identify which patients with VTE were at an increased risk for subsequent cancer [8]. Our score helped to identify the most common sites of cancer according to gender and age subgroups. Moreover, this score has been externally validated but only after replacing one variable, and this implies a limitation [9].

The RIETE (Registro Informatizado de Enfermedad TromboEmbólica) registry is an ongoing, multicenter, international registry of consecutive patients with objectively confirmed acute VTE (ClinicalTrials.gov identifier: NCT02832245). RIETE registry has generated manuscripts evaluating other outcomes as bleeding or mortality, and risk factors for these outcomes [10–13]. The aim of the current study was to validate the prognostic score using a subsequent cohort of unprovoked VTE patients in the RIETE Registry.

## Patients and methods

### Inclusion criteria

Inclusion criteria: acute symptomatic, objectively proven VTE included in the RIETE Registry. Cases: Patients diagnosed with cancer from 30 days to 24 months after unprovoked VTE. Controls: Patients with no previous cancer followed-up for at least 12 months after unprovoked VTE. Exclusion criteria: 1) Previously known cancer, 2) No previous cancer, but follow-up <12 months, 3) Patients not receiving anticoagulant therapy, 4) Patients participating in a clinical trial with a blinded therapy. In all patients oral or written consent was obtained (according to local Ethics Committee requirements of each center), and when minors were included we obtained consent from parents of guardians (Authorization of clinical research ethics committee Germans Trias i Pujol and Institut Catalá de la Salud. 05122006). Data were recorded from each participating hospital and submitted to a coordinating center through a secure website. Each patient was assigned with a unique identification number to maintain patient confidentiality, and data quality was regularly monitored electronically.

### Study design

For external validation we considered RIETE Registry patients from a different cohort of patients that derivation and internal validation [8], including patients recruited from January 2014 to October 2016. We performed a nested case control study within a cohort of patients included in the RIETE Registry [14]. For diagnosing cancer, histological confirmation was always required. Patients diagnosed with cancer beyond the first 30 days after unprovoked VTE were identified as cases, and those with no cancer with a follow-up at least 12 months after unprovoked VTE were identified as controls. Patients with a follow-up <12 months were not considered for the study.

### Baseline variables and follow-up

Patients enrolled in the RIETE registry had data collected from around the time of VTE diagnosis that included but was not limited to: age; sex; weight; presence of coexisting conditions such as chronic heart or lung disease; recent (<30 days before VTE) major bleeding; extent of the venous thrombosis (distal thrombosis was thrombosis confined to the infra-popliteal veins); clinical signs and symptoms on admission; laboratory results at baseline. Creatinine clearance levels were measured according to the Cockcroft and Gault formula [15]). Anemia was defined as hemoglobin levels <13 g/dL for men and <12 g/dL for women.

Unprovoked VTE was defined as those occurring in the absence of risk factors for VTE, including cancer, recent immobility (defined as non-surgical patients assigned to bed rest with bathroom privileges for >4 days in the 2 months before VTE diagnosis); surgery (defined as those who had undergone major surgery in the 2 months before VTE), hormonal therapy, recent travel, pregnancy or puerperium.

Patients were managed according to the current clinical practice of each participating hospital, and type, dose, and duration of different treatments were recorded. During each visit, signs or symptoms suggesting cancer, ‎symptomatic VTE or major bleeding were noted. In patients with suspected malignancy, the attending physicians decided which diagnostic tests were performed.

### Statistical analysis

We compared baseline characteristics of patients with and without cancer using chi-square tests for categorical variables and non-parametric rank tests for continuous variables. From a practical point of view, and taking into account that most of cancers are diagnosed within the first year after unprovoked VTE, we tried to validate our score for occult cancer at 12 and 24 months (Table 1). As we applied the score in unprovoked VTE, previous surgery have been excluded from table due to any patient achieve this condition. We estimated the cumulative incidence of cancer using the Kaplan–Meier technique. We assessed the discriminative power by calculating the area under the receiver-operating characteristic (ROC) curve, performing a non-parametric test of the equality of the areas under the ROC curves. We determined the goodness-of-fit of the score points for each score in a logistic regression model using Pearson’s chi-square test.

**Table 1:** Risk prediction score for unprovoked venous thromboembolism (modified from Jara-Palomares et al. [8]).

Underlying conditions	Points
Male gender	**+ 1**
Age >70 years	**+ 2**
Chronic lung disease	**+ 1**
Anaemia	**+ 2**
Platelets ≥350x10^6^/mm^3^	**+ 1**
Prior VTE	**- 1**
**Classification**	
Low risk	≤2
High risk	≥3

Anaemia was defined as: Haemoglobin levels <12 g/dL in women, <13 g/dL in men.

Abbreviations: VTE, venous thromboembolism.

As we analysed patients with unprovoked VTE only, postoperative VTE has been deleted from score

As we applied the score in patients with unprovoked VTE, previous surgery has been excluded from the Table since no patient achieved this condition. When we built the prediction model, chronic lung disease was found to be an independent predictor for occult cancer [8]. Other authors found tobacco smoking to be an independent predictor [16]. In the current study, current smoking was also collected, and we analyzed the impact on the C-statistic if we consider current smoking instead of chronic lung disease. For the statistical analysis we used the IBM SPSS Statistics program (version 19; SPSS Inc., Chicago, IL), and a two-sided p<0.05 was considered to be statistically significant.

## Results

### Study sample

**Fig 1:**
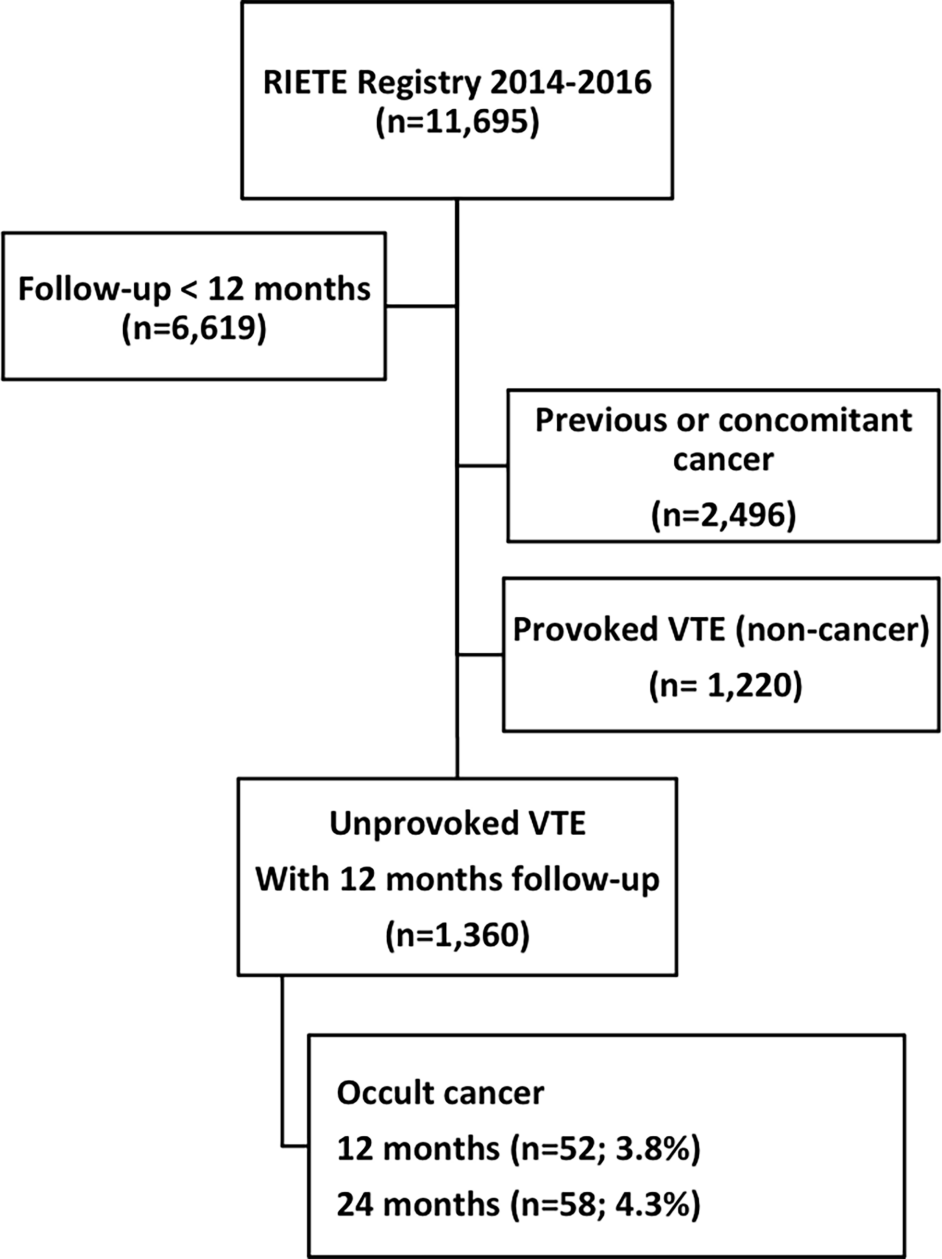
Flowchart patients.

From January 2014 to July 2016, 11,695 VTE patients were recruited. Of them, 2,496 had previous or concomitant cancer, 1,220 had provoked VTE (with no cancer) and 6,619 were excluded because they were not followed up for at least 12 months (Fig 1). There were 1,360 eligible patients. Mean age was 66±16 years, and 57% were male. Patients who subsequently were diagnosed with cancer were significantly older, and more likely to have anemia or platelet count ≥350,000 x 1,000/mm^3^ at baseline than those without cancer. Among 34 men with cancer, the most frequent cancer sites were the lung (24%), prostate (18%), hematologic (18%) and colorectal (15%). Among 24 women with occult cancer, the most frequent sites were hematologic (17%), colorectal (17%), uterine (13%), breast (8%), pancreas (8%) and ovarian (8%). (Clinical characteristics are in Table 2)

**Table 2:** Clinical characteristics of patients with vs. without occult cancer.

Patients, n	Total (n = 1,360)	Occult cancer (n = 58)	No cancer (n = 1,302)	p
**Clinical characteristics,**				
Gender (male), n (%)	772 (56.8)	34 (58.6)	738 (56.7)	0.77
Age > 70 years, n (%)	677 (50)	39 (67.2)	638 (49.2)	< 0.05
Weight (kg), mean (SD)	79.3 (16.2)	75.5 (13)	79.5 (16.3)	0.06
**Co-morbid diseases, n (%)**				
Chronic lung disease	169 (12.4)	11 (19)	158 (12.1)	0.12
Chronic heart failure	62 (4.6)	2 (3.4)	60 (4.6)	0.68
Prior VTE	220 (16.2)	11 (19)	209 (16.1)	0.56
**Laboratory findings, n (%)**				
Anaemia	282 (20.9)	20 (34.5)	262 (20.2)	< 0.05
Leucocytes >11,000 x 1,000/mm^3^	335 (24.8)	11 (19)	324 (25)	0.3
Platelet count ≥350,000 x 1,000/mm^3^	76 (5.6)	7 (12.1)	67 (5.3)	< 0.05
Raised fibrinogen levels	349 (38.5)	17 (42.5)	332 (38.3)	0.6
**Initial VTE presentation, n (%)**				0.25
• DVT	565 (42.2)	26 (46.4)	539 (42)	
• Pulmonary embolism	540 (40.3)	17 (30.4)	523 (40.8)	
• DVT / pulmonary embolism	234 (17.5)	13 (23.2)	221 (17.2)	
Proximal DVT	665 (90)	35 (89.7)	630 (90)	0.96
Bilateral DVT	9 (4.3)	1 (3)	8 (4.5)	0.9
Upper extremity DVT	46 (5.4)	0 (0)	46 (5.7)	0.13

Abbreviations: SD, standard deviation; VTE, venous thromboembolism; DVT, deep vein thrombosis.

### 12 months score prediction

In all (n = 1,360), 52 patients (3.8%; 95%CI: 2.9 to 5.0%) were diagnosed with cancer within the first 12 months. Among 905 patients scoring ≤2 points (67%), 22 (2.4%; 95% CI 1.5 to 3.7%) had occult cancer. Among 455 patients scoring ≥3 points, 30 (6.6%; 95% CI 4.5 to 9.3%) were subsequently diagnosed with cancer (hazard ratio 2.8; 95% CI 1.6 to 5). C-statistic was 0.63 (95% CI 0.55 to 0.71).

### 24 months score prediction

**Fig 2:**
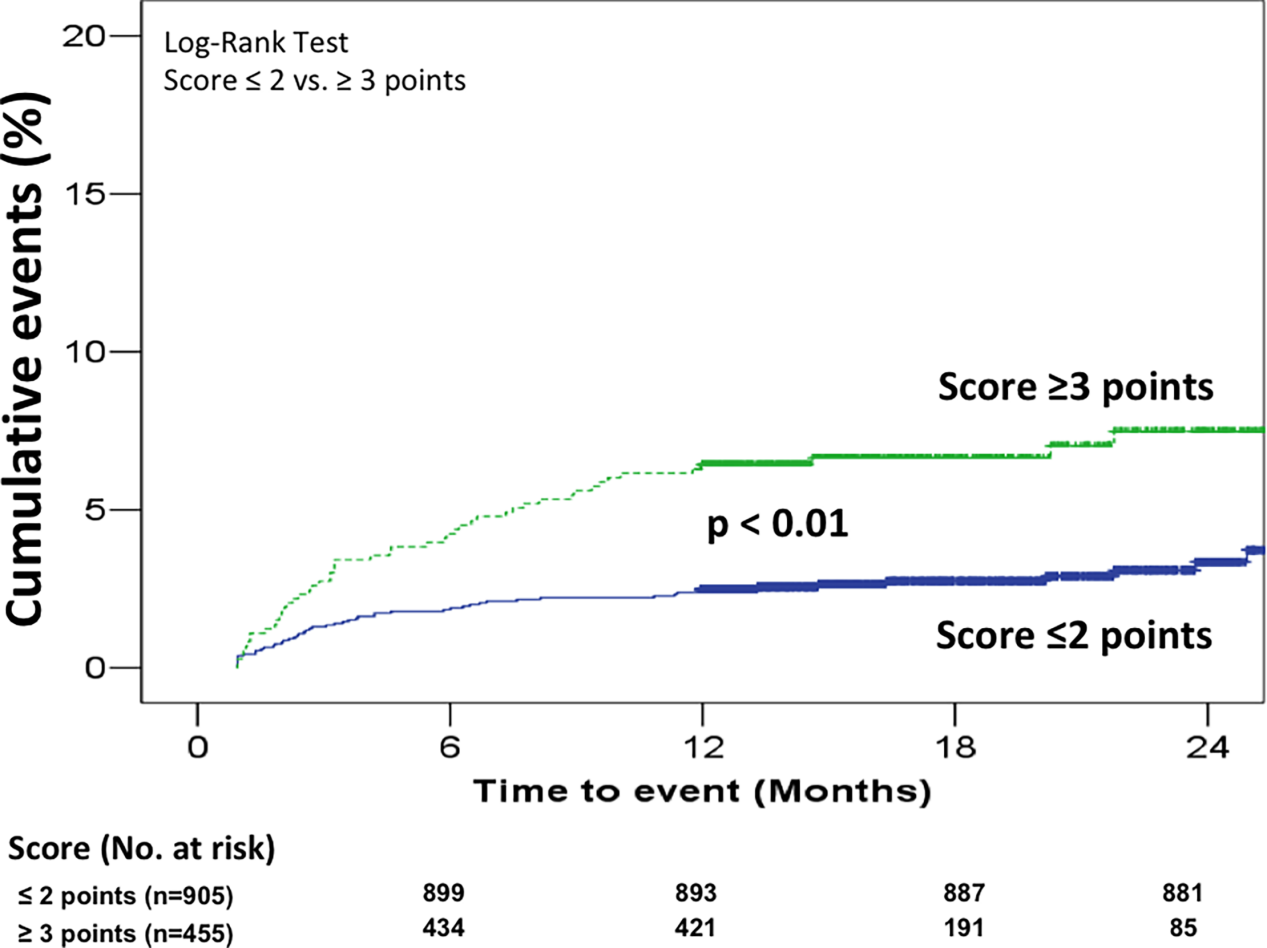
Cumulative incidence of occult cancer over 24 months attending score (≤2 vs. ≥3 points). Time-to-event data.

At 24 months, 58 of 1,360 eligible patients (4.3%; 95%CI: 3.3 to 5.5%) were diagnosed with cancer. Among 905 patients scoring ≤2 points, 26 (2.9%; 95%CI: 1.9 to 4.2%) had subsequent cancer. Among 455 patients scoring ≥3 points, 32 (7%; 95%CI: 4.9 to 9.8%) had cancer (hazard ratio 2.6; 95% CI 1.5 to 4.3; p<0.01) (Fig 2). C-statistic was 0.61 (95% CI, 0.54 to 0.69).

### Chronic lung disease vs. current smoker

Of 169 patients with chronic lung disease 11 (6.5%) had occult cancer. Of 218 current smokers 11 (5%) had occult cancer. At 24 months, using the original prediction model, C-statistic was 0.61 (95% CI, 0.54 to 0.69). After replacing chronic lung disease for current smoking, the C-statistic was 0.62 (95% CI, 0.54 to 0.69) (Table 3).

**Table 3:** Multivariable analysis replacing chronic lung disease for current smoke.

24 months validation	β	Odds ratio	Lower 95% CI	Upper95% CI	p
Male gender	.17	1.19	.64	2.22	.59
Age >70 years	.59	1.8	.88	3.65	.11
Current smoke	.26	1.3	.61	2.78	.5
Anaemia	-.07	.93	.48	1.81	.83
Platelets ≥350x10^6^/mm^3^	1.01	2.76	1.05	7.23	.04
Previous VTE	0.1	1.1	.52	2.33	.80

24 months C-statistic: 0.62 (95% CI, 0.54–0.69)

Anaemia was defined as: Haemoglobin levels <12 g/dL in women, <13 g/dL in men.

Abbreviations: CI, confidence intervals; DVT, deep vein thrombosis; VTE, venous thromboembolism.

## Discussion

Our findings, obtained from a large sample of consecutive patients with unprovoked VTE, validated our recently reported score. Our prognostic score contains 6 easily available variables that help to identify patients at increased risk for occult cancer. Less than one third of these patients were considered to be at high risk, and the proportion of patients with cancer in this subgroup was higher than in the low risk subgroup: 7% vs. 2.9% (hazard ratio 2.6; 95% CI 1.5–4.3). Some variables in the score are consistent with previous studies that evaluated the risk for occult cancer in VTE [16, 17]. In 2004, Piccioli et al. identified patient’s age as predictor for occult cancer in a subgroup analysis of a randomized controlled trial [18]. In 2013, Ferreyro et al. built a prognostic score based on 32 of 540 patients with VTE who subsequently were diagnosed with cancer [17]. Variables associated with an increased risk for occult cancer included prior VTE, no recent surgery and Charlson score ≥2. This score has not been then validated, and their results show large confidence intervals, thus suggesting that the data are not robust, mostly due to few events. In 2016, Ihaddadene et al. published post-hoc, pre-defined analyses of the SOME trial [16]. The study enrolled 854 patients, of whom 33 (3.9%; 95% C.I. 2.8 to 5.4) received a diagnosis of cancer at 1-year follow-up. Age, prior provoked VTE and smoking habit were identified as predictors of occult cancer in patients with first unprovoked VTE. Recently, our score was been validated in a post hoc analysis from the MVTEP study [9, 19]. In this study, authors validated the score, but the main limitation was that they did not collect data on chronic lung disease and to obtain validation they replaced tobacco instead of chronic lung disease.

Previous studies failed to demonstrate any benefit for extended screening, with a lower than expected prevalence of occult cancer [19–22]. The final step in screening is to show preventable deaths and years of life gains. For example, in colon cancer screening if a birth cohort of 4 million were offered screening at recommended intervals, 31,500 deaths would be prevented, which implies to screen 127 patients to prevent 1 death [23]. Therefore, a strategy to identify a high-risk population would be meaningful. The PROSPR consortium is a multidisciplinary approach to improve the screening in any site (i.e. breast or colon cancer screening) [24–26]. They suggest a five-step way to evaluate and improve screening. The first step is to identify the recruitment of eligible patients, and this is the step where we are currently placed. After focusing on a high-risk population we could evaluate to go to the second step, because we need to avoid over-screening.

Our study has several strengths. First, we included prospective data collected within a multicenter international registry and the sample for this validation was high. Second, the score takes into account easily available patient characteristics, (which had shown an important role on this topic). Third, we calculated the C-statistic after replacing former or current smoking instead of chronic lung disease, with similar results (0.61; 95% CI, 0.54–0.69 vs. 0.62; 95% CI, 0.54 to 0.69). We assume that it not implies a validation of this replacement but it could be an interesting exploratory topic. On this way, Bertoletti et al. replaced this variable to validate score [9].

Our study also has a number of limitations. This is a retrospective analysis from a registry where patients were recruited consecutively, which could imply selection bias. The results of this study should encourage other researchers to externally validate this score in prospective studies, and to use it to select a target population that would benefit more. Second, the area under the curve of our prognostic score was mild, but allowed us to identify a high-risk population using score ≥3 points. Finally, RIETE does not have a standardized protocol for the follow-up of patients, and we do not have data about additional tests that were performed on patients. In the same way, we do not dispose of data about false positive results, economical or psychological implications secondary to these complementary studies. This approach is crucial to implement screening process, and should be aboard in a clinical trial.

## Conclusion

We validated a prognostic score for occult cancer in patients with unprovoked VTE, although prospective validation is needed. According to PROSPR consortium, once we have identified a high-risk population we could go to the second step in the screening process, which implies to select a screening strategy.
